# Tracking Rift Valley fever: From Mali to Europe and other countries, 2016

**DOI:** 10.2807/1560-7917.ES.2019.24.8.1800213

**Published:** 2019-02-21

**Authors:** Christelle Tong, Emilie Javelle, Gilda Grard, Aissata Dia, Constance Lacrosse, Toscane Fourié, Patrick Gravier, Stéphanie Watier-Grillot, Renaud Lancelot, Franck Letourneur, Frédéric Comby, Martin Grau, Lionel Cassou, Jean-Baptiste Meynard, Sébastien Briolant, Isabelle Leparc-Goffart, Vincent Pommier de Santi

**Affiliations:** 1 French Armed Forces Centre for Epidemiology and Public Health (CESPA), Marseille, France; 2 Laveran Military Teaching Hospital, Marseille, France; 3 French Armed Forces Biomedical Research Institute (IRBA), National Reference Centre for Arboviruses, Marseille, France; 4 Unité des Virus Émergents (UVE), Aix-Marseille Université – IRD 190 – Inserm 1207 – IHU Méditerranée Infection, Marseille, France; 5 International Centre of Agricultural Research for Development (CIRAD), Animals, Health, Territories, Risks and Ecosystems Unit (ASTRE), Montpellier, France; 6 UMR ASTRE, Univ. Montpellier, CIRAD, INRA, Montpellier, France; 7 French Military Health Service, 7th Medical Unit, Lyon, France; 8 French Military Health Service, 10th Medical Unit, Laudun, France; 9 French Military Health Service, 18th Medical Unit, Fréjus, France; 10 French Military Health Service, 11th Medical Unit, Toulouse, France; 11 French Armed Forces Biomedical Research Institute (IRBA), Marseille, France; 12 UMR VITROME, Aix-Marseille Université, IRD, AP-HM, SSA, VITROME, IHU-Méditerranée Infection, Marseille, France

**Keywords:** Rift Valley fever, Imported viral disease, Whole blood, vector-borne infections, Mali, Service members

## Abstract

On 16 September 2016, the World Health Organization confirmed a Rift Valley fever (RVF) outbreak in Niger. Epidemiological surveillance was reinforced among the French Armed Forces deployed in Niger and bordering countries: Chad, Mali and Burkina Faso. On 26 October, a probable case of RVF was reported in a service member sampled in Mali 3 weeks earlier. At the time the result was reported, the patient was on vacation on Martinique. An epidemiological investigation was conducted to confirm this case and identify other cases. Finally, the case was not confirmed, but three suspected cases of RVF were confirmed using serological and molecular testing. RVF viral RNA was detectable in whole blood for 57 and 67 days after onset of symptoms for two cases, although it was absent from plasma and serum. At the time of diagnosis, these cases had already returned from Mali to Europe. The infectivity of other arboviruses in whole blood has already been highlighted. That RVF virus has been detected in whole blood that long after the onset of symptoms (67 days) raises the question of its potential prolonged infectivity. Because of exposure to tropical infectious diseases during deployment, military populations could import emerging pathogens to Europe.

## Background

Rift Valley fever (RVF) is an arbovirosis affecting both domestic and wild ruminants, especially sheep, cattle and goats, as well as humans (zoonosis) [1]. RVF is caused by a mosquito-borne virus of the family Phenuiviridae and genus Phlebovirus. Its impact can be considerable, with large outbreaks reported in the past: 200,000 human cases and 600 deaths in Egypt in 1976, and 230 deaths among 747 human cases in Sudan in 2007 and 2008 [2,3]. RVF virus (RVFV) is widespread in Africa, with spillover to the Comoros Archipelago (including Mayotte), Madagascar, Saudi Arabia and Yemen. So far, Europe has been free from active viral circulation. However, competent vectors have been identified in several European countries and RVFV introduction is a real concern [4].

Humans are mainly infected by contact with body fluids and tissues of infected animals (live or dead); dietary exposure is also suspected (e.g. raw or unpasteurised milk). They can also be infected by mosquito bites. Mosquitoes of the genus *Aedes* and *Culex* are considered to be the most competent vectors to transmit RVFV. The primary foci of RVF epidemics are mainly triggered by heavy rainfall episodes, when vectors are abundant [4-6]. RVF infection in humans is generally asymptomatic [7]. Symptomatic forms are mostly benign (dengue-like illness), occur after a 2–6-day incubation period and last less than a week [8,9]. However, severe forms may be observed, with complications such as encephalitis (up to 5% of cases, up to 60 days after the onset of symptoms), haemorrhagic fever (less than 1%) or retinitis (up to 20%) [10-12]. Viraemia of RVFV spans the acute febrile phase of the disease, i.e. 3 or 4 days [7].

The French Armed Forces (FAF) have been deployed in Africa’s Sahel region, including Niger, Mali, Chad and Burkina Faso, since 2014. Considering the risk of arboviral infections during such deployment, unexplained fever and dengue-like syndrome have been under mandatory epidemiological surveillance in the FAF since 2004. In addition, since January 2016, dried blood spot samples have been routinely collected on blotting paper for any service member presenting an undiagnosed fever. Collected samples are sent to the French National Reference Centre (CNR) for Arboviruses in Marseille, France, for serological testing and viral RNA detection.

## Outbreak detection

On 16 September 2016, the World Health Organization confirmed an RVF epidemic on the Western Niger border with Mali, in the Tahoua region [13]. The outbreak occurred in the population of transhumant livestock farmers, with 399 human cases and 33 reported deaths [14]. An epizootic RVF outbreak was also reported among livestock during the same period [13].

This worrisome RVF epidemic in Niger led the French Military Health Service to enhance RVF prevention starting on 23 September 2016. Information about the disease was communicated to field military medical staff and service members. Contact with local animals and consumption of local animal products were strictly forbidden. A reminder of the epidemiological and microbiological surveillance procedures was given.

On 26 October 2016, the CNR for Arboviruses reported to the French Armed Forces Centre for Epidemiology and Public Health (CESPA) in Marseille the detection of RVFV RNA in one blotting paper blood sample using reverse transcription-PCR (RT-PCR) [15]. The patient was a French service member deployed from June to October 2016 in a small village called Abeïbara, in the Kidal region in north-eastern Mali. Three weeks before the alert, on 6 October, during a short stay in Gao, Mali, he had presented a dengue-like illness that lasted 48 hours, without complications. A blood sample was taken on blotting paper on 7 October. He returned to France on 14 October after a 3-day stay on Crete, Greece, and went on leave on Martinique, French West Indies, on 22 October. By the time the alert was issued, most of the service members deployed in Abeïbara had returned to France and were on leave.

We report here the epidemiological and biological investigations conducted in order to confirm the described case, search for additional suspected cases and identify the RVFV exposure factors.

## Methods

### Epidemiological investigations

#### Probable case confirmation

Laboratory diagnosis confirmation of the initial reported case was the first step of the investigation, as the alert relied on a single RT-PCR assay performed on a dried blood sample. The patient was defined as a probable case. Considering that RVFV RNA has previously been detected up to 20 days after onset of illness in whole blood and up to 4 months after onset in semen [16,17], the patient, who was on leave in the French West Indies (Martinique) 20 days after onset of illness, was presumed to still be viraemic. The probability of RVFV being imported to Martinique, where competent vectors are abundantly present, was considered non-negligible. Therefore, the patient was urgently addressed to a local military health centre for clinical evaluation. Individual protection measures against mosquito bites were implemented. Blood samples were taken and sent to continental France for analysis.

A confirmed case was defined as a patient who had laboratory confirmation of a recent RVF infection.

#### Case finding

The second step was to identify suspected cases. They were defined as any French service member presenting fever ≥ 38.5 °C (the threshold defined for fever in the FAF Epidemiological surveillance system, considering hot climate in tropical areas and physical activities during deployment), associated with headache or retro-orbital pains or arthralgia or myalgia or rash, between 1 September and 15 October 2016 in Mali. An extended search of suspected cases was initiated using data from various sources: epidemiological surveillance data focusing on dengue-like syndrome and unexplained fever, samples received by the CNR for Arboviruses, and the list of patients seen for fever provided by military medical staff deployed in Mali. For each suspected case, a medical doctor administered a face-to-face questionnaire and a venous blood sample was collected. The questionnaire included items about health events and exposure to RVF during military deployment and more precisely during the 10 days (enlarged incubation period) preceding any symptoms that could be related to RVF (isolated fever or dengue-like syndrome) (Supplement).

#### Cross-sectional study

The purpose of the third and last step was to identify cases of RVF who did not seek medical care and/or cases of asymptomatic RVF infection, and to estimate exposures to known RVF risk factors. A cross-sectional study was conducted among the 187 service members of the military contingent deployed in Abeïbara. This investigation took place in France on 16–17 November 2016, after the return of military units. The inclusion criterion was being a member of the contingent who was present in Abeïbara during September or October 2016 (end of the deployment of this contingent in Mali). For each subject included, the same anonymous questionnaire used for suspected cases was self-administered during a collective meeting, and whole blood and plasma were collected for viral diagnosis.

### Microbiological investigations

All the microbiological investigations were performed by the French CNR for Arboviruses in Marseille, France. The criterion for a laboratory confirmation of a recent RVF infection was the detection of RVF RNA in any specimen and/or presence of RVFV IgM antibodies. Serological investigations were performed using MAC-ELISA as previously described [18] and indirect ELISA for IgG detection. Plasma from suspected cases and from subjects included in the cross-sectional survey were analysed with the following IgG/IgM serological tests: RVFV, dengue virus (DENV), West Nile virus (WNV), Zika virus (ZIKV) and chikungunya virus (CHIKV).

We screened for RVFV RNA in plasma, whole blood and semen when available, by RT-PCR using two different TaqMan assays targeting distinct viral genome sequences [15,19]. Sequencing and viral isolation on Vero and C6/36 cell lines were attempted for all PCR-positive blood samples. 

### Ethical statement

All participants of the cross-sectional study were volunteers and received information about the study and the disease. Each confirmed case received care adapted to their state of health provided by the French military health service. Informed and signed consent for the use of their data were obtained from each confirmed case. Considering the possibility of late complications for RVF (encephalitis) cases and according to French regulation in case of an outbreak with immediate public health threat (RVFV importation), no ethical approval was required.

## Results

### Probable case investigation

On 28 October, 2 days after the alert, the probable case was examined at a French military health centre on Martinique and was asymptomatic. In the interview, he did not recall any contact with live or dead animals but mentioned nocturnal mosquito bites. Temporary individual protection measures against mosquito bites were implemented during his stay in Martinique. Another blood sample collected one week later was PCR-negative as well as IgM- and IgG-negative for RVFV, therefore invalidating the case. The false positivity of this case can be attributed to a lack of PCR specificity carried out on a dried blood sample [15]. A second RT-PCR assay targeting a distinct RVFV sequence [19] was retrospectively performed on the first blotting paper and was negative. This RT-PCR was then implemented in the CNR laboratory in a systematic way. 

According to the patient, several other military personnel of the Abeïbara contingent had presented fever and myalgia during the same period in Mali. A search of suspected cases was conducted based on his statements.

### Investigation of suspected cases

No case of unexplained fever or dengue-like syndrome had been reported to the epidemiological surveillance by the FAF in Mali from September to early October 2016 (end of mission for the Abeïbara contingent). Active research identified six suspected RVF cases in Mali from September to October 2016. At the time of the alert, one was still deployed in Mali and five had completed their mission and returned to France after a 3-day stay in Crete, Greece. Of the latter, two stayed in France, and the three others were on leave in Ukraine, Ireland and Nepal (Figure). Finally, three of the six suspected cases were confirmed as RVF cases several weeks after the onset of symptoms (Figure).

**Figure:**
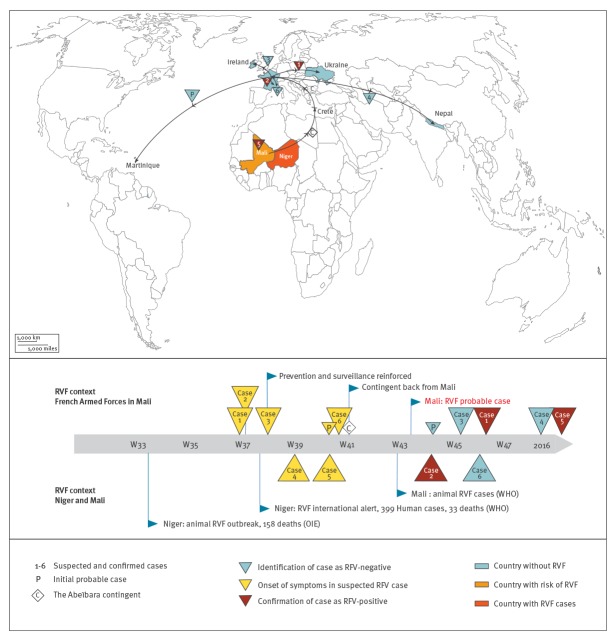
Travel from Mali and stays in other countries of suspected, probable and confirmed Rift Valley fever cases, French Armed Forces, Mali, 2016–2017 (n = 6)

OIE: World Organisation for Animal Health; RVF: Rift Valley fever; WHO: World Health Organization.

### Biological and clinical features of the confirmed cases

The first confirmed case (Case 1, Figure and Table 1) was a 28-year-old man, deployed in Mali from June to early October 2016. During the RVF incubation period, he stayed in Abeïbara, had several direct contacts with live ruminants (camels and goats), consumed goat meat purchased from a livestock farmer, and reported between one to two mosquito bites per day. On 12 September, he presented fever (> 39 °C), headache with retro-orbital pain, myalgia, and photophobia. The symptoms resolved within 24–48 hours, without complications. The patient left Mali 24 days after the onset of symptoms, spent 3 days in Crete, several days in the south of France, then several weeks in Ukraine and returned to France in mid-November. On his return, he was hospitalised due to the persistence of intermittent headaches with photophobia. Meningoencephalitis was excluded in normal clinical examinations, cerebral magnetic resonance imaging (MRI) with no signal abnormality and cerebrospinal fluid parameters. Ophthalmological examinations revealed normal visual acuity and two old peripheral lesions from a chorioretinal focus to the right eye, without any evolution 3 months later. Laboratory testing revealed the presence of IgM and IgG against RVFV. RT-PCRs were negative in serum, plasma and semen at 67 days after the onset of symptoms, but viral RNA was still detected in whole blood, which was confirmed by partial viral sequencing (derived 683 nt sequence GenBank: BankIt2148117 RVF41318 MH880842). It was not possible to obtain a larger sequence of viral RNA. In addition, viral isolation attempts on VERO and C6/36 cell lines remained unsuccessful. The patient also had positive *Toxoplasma gondii* serology (IgG without IgM), which may explain the chorioretinal scars.

**Table 1:** Timeline of confirmed Rift Valley fever cases after exposure in Mali, French Armed Forces, 2016–2017 (n = 3)

	Case 1	Case 2	Case 5
Exposure during incubation period			
Location	Abeïbara	Abeïbara	Gao
Contact with ruminants			
Direct/touching	−	+	−
Indirect/proximity	+	+	−
Mosquito bites	> 10 bites/day	1–2 bites/day	Not observed
Acute symptoms			
Fever > 38.5 °C	+	+	+
Headache	+	+	+
Retro-orbital pain	+	−	+
Photophobia	+	−	+
Myalgia	+	+	+
Arthralgia	−	+	+
Diarrhoea	−	−	+
Date of onset	12 Sep 2016	13 Sep 2016	05 Oct 2016
Location	Abeïbara	Abeïbara	Gao
Duration	3 days	10 days	2 days
Locations after febrile stage	Crete, France, Ukraine	Crete, South of France	Mali
RVFV serology			
Sample date	18 Nov 2016	03 Nov 2016	22 Mar 2017
Results	IgM- and IgG-positive	IgM- and IgG-positive	IgM- and IgG-positive
RT-PCR			
Whole blood	Positive	Positive	NA
Day after onset	67 days	57 days	NA
Plasma	Negative	Negative	Negative
Semen	Negative	Negative	NA
Viral sequence	683 nt	683 nt	NA
Culture	Negative	Negative	NA

NA: not available; RVFV: Rift Valley fever virus.

The second case (Case 2, Figure, Table 1) was a 38-year-old man. He was deployed in Mali from June to the end of September 2016. During the incubation period, he stayed in Abeïbara and reported proximity with goats and camels without direct contact and more than 10 mosquito bites per day, occurring night and day. On 13 September, he presented fever (> 39 °C) associated with headache, myalgia and arthralgia. He was hospitalised for 4 days and received symptomatic treatment. The symptoms lasted 10 days, without complications. He left Mali 15 days after the onset of symptoms, spent 3 days in Crete and stayed in the south of France. Normal clinical examination, including ophthalmological examination, was reported on 2 November 2016. Semen and blood samples were collected 57 days after the onset of symptoms (9 November). Plasma was positive for anti-RVFV IgM and IgG, PCRs were negative in semen and plasma, while viral RNA was still detected in whole blood as was confirmed by partial viral sequencing. The derived 683 nt sequence (GenBank BankIt2148117 RVF41125 MH880841) differed by only 1 nt from the sequence obtained from Case 1. As for Case 1, viral isolation attempts remained unsuccessful.

The third case (Case 5, Figure, Table 1) was a 54 years-old man. He was deployed in Mali from August to December 2016 and returned to France in January 2017. During the incubation period, he stayed in Gao, had no contact with ruminants and did not report mosquito bites. On 5 October, he presented fever (> 39 °C), headache, photophobia, arthralgia and myalgia for 2 days. Two dried blood samples on blotting papers were collected during the symptomatic phase. Serologic tests and RT-PCR were negative for the first sample and insufficient blood quantity led to uninterpretable laboratory results for the second sample. A late sample collected in March 2017 was positive for anti-RVFV IgM and IgG, but PCRs remained negative. The patient also complained of long-lasting headaches with normal cerebral and ophthalmological investigations.

Cases 1 and 2 were kept under personal anti-vector protective measures and were excluded from blood donation until clearance of their viraemia, 100 days after the onset of symptoms.

### Cross-sectional study

Of the 187 service members, 99 (53%) were included in the cross-sectional study. Fifteen participants reported a fever episode that had led 12 of them to seek medical care (Table 2). None of these fever cases was reported to the epidemiological surveillance system. Retrospectively, 14 could be considered suspected RVF cases. Among those, six reported being symptomatic between 7 and 15 October, suggesting grouped cases. No other recent arbovirus infections were observed (absence of specific IgM) (Table 2). Only one participant, who did not report any symptoms, had a positive RVFV IgG result, suggesting a previous exposure to the virus. In addition, 26 participants revealed a previous exposure to a flavivirus (dengue virus or/and WNV or/and ZKV) and 20 to an alphavirus (CHIKV). Among these subjects, three were positive for both alphavirus and flavivirus IgG and one for both flavivirus and RVFV IgG.

**Table 2:** Seroprevalence of arboviruses, symptoms and exposure to Rift Valley fever virus reported by the military contingent deployed in Abeïbara, Mali, in September and October 2016, French Armed Forces (n = 99)

Participant characteristics	n	%
Reported symptoms		
Feeling feverish	15	15.2
Headache	29	29.3
Retro-orbital pain	6	6.1
Vision disorders	3	3.0
Arthralgia	16	16.2
Myalgia	21	21.2
Diarrhoea	18	18.2
Retrospectively classified as suspected cases^a^	14	14.2
Seeking care for fever	12	12.2
Positive arbovirus serology		
IgM RVFV	0	0.0
IgG RVFV	1	1.0
IgM Flavivirus (dengue, WNV, ZKV	0	0.0
IgG Flavivirus (dengue, WNV, ZKV)	26	26.3
IgM Alphavirus (CHIKV)	0	0.0
IgG Alphavirus (CHIKV)	20	20.2
Exposure to RVFV		
Direct contact with ruminants^b^	35	35.4
Contact with dead ruminants^b^	20	20.2
Participation in the slaughter of a goat	28	28.3
Cleaning or sleeping in a room where animals were kept	47	47.5
Care of a wounded person	6	6.1
Consumption of raw milk	3	3.0
Mosquito bites	84	84.8

CHIKV: chikungunya virus; RVFV: Rift Valley fever virus; WNV: West Nile virus; ZKV: Zika virus.

^a^ According to the definition of suspected cases: French service member who presented fever > 38.5 °C associated with headache or retro-orbital pains or arthralgia or myalgia or rash between 1 September and 15 October 2016.

^b^ The implicated ruminants were goats, zebu cattle and, in rare cases, camels.

Among the included subjects, 39% encountered goats, zebu cattle and donkeys inside the military camp, 35% had direct contact with live animals, and 20% declared they had touched or handled dead animals (mostly goats and zebu cattle), while 28% had participated in animal slaughter (mostly goats or zebu cattle). Three service members reported the consumption of raw goat or camel milk. In addition, 85% reported mosquito bites, of whom 17% declared more than five bites per day. Bites were predominantly nocturnal in 81% of cases, but could also occur during the day. During September and October 2016, most of the interviewed service members (72%) consumed local meat, mainly zebu and goat meat. 

### Control measures

Contact with local animals and consumption of local animal products were strictly forbidden for French service members. Individual protection measures against mosquito bites were reinforced. No other cases of RVF have been reported since this outbreak in the FAF deployed in Mali.

## Discussion

### Rift Valley fever in Mali

RVF virus has probably been circulating in Mali for a very long time [20]. Sporadic human cases have been observed [17,21]. A recent serological study conducted among cattle in Mali revealed RVFV circulation among ruminants, with a seropositivity prevalence for RVF of 10.0% among bovines in Kidal and 15.8% in Gao [22]. In March 2017, considering the RVF outbreak in Niger and the transhumance patterns of livestock farmers, experts from the Food and Agriculture Organisation of the United Nations took the view that RVF was likely/very likely (a 66–99% chance range) to occur in Mali during the vector season [23]. In our study, we confirmed three human cases of RVF in French service members who occurred in Mali at the end of the rainy season in 2016. No sequelae were observed except persistent headaches for months in two of them. These findings were made by chance, thanks to a non-confirmed false-positive test. 

The small number of confirmed cases in our study did not allow us to identify risk factors for RVF through statistical analyses. In addition, face-to-face questionnaires may have introduced a bias in the responses on risky behaviours service members did not admit to. The case in Gao (Case 3) was probably infected by a mosquito bite. The other two cases were exposed in Abeïbara, an isolated location between the city of Kidal and the border with Algeria with a hot desert climate and less than 50 mm of rainfall per year. The presence of wells in the village allows the local population to raise ruminants (mainly goats, zebu cattle and camels). Moderate rainfall from mid-July to mid-August probably promoted the emergence of *Aedes* mosquitoes, which explains the widespread exposure to mosquito bites reported by service members in our study [24]. RVFV exposure for Cases 1 and 2 could be linked to direct contact with ruminants, particularly during the slaughter of infected goats, or to mosquito bites.

Because of insecure conditions and repeated attacks around the military camp, entomological investigations and biological analyses on local livestock could not be performed. Regarding RVF in West Africa, this local transmission in a desert area may be the result of either the introduction of sick animals by nomadic herders or transovarian transmission of the virus in some mosquito species, such as *Aedes vexans*, whose infected eggs survive desiccation during the dry season and hatch infected imagos when temporary ponds are flooded by rainfall [25,26].

### Service members: travellers exposed to a specific infectious risk

During field operations, service members are deployed in rural, remote, and non-tourist areas, close to the local populations they need to protect, and are therefore exposed to the same health risks. Scarcity of water resources in the Sahel desert explains the proximity of service members to ruminants because both use the same water sources (35% had direct contact with ruminants) and the increased risk of exposure to zoonoses. The large proportion (28%) of service members involved in the slaughter of goats was more surprising. Although slaughter and consumption of local meat is forbidden in the FAF, it has been observed in isolated military units with strong logistic constraints [27]. In addition, slaughtering occurred before the implementation of RVF preventive measures and the risk of RVFV exposure could have been underestimated at that time. Exposure to vector-borne diseases, especially arboviroses, appears to be high in the military population. Among the subjects included in the cross-sectional study, 20% and 26% presented serological evidence of exposure to, respectively, alphaviruses and flaviviruses. In addition, 84% reported mosquito bites during deployment in Abeïbara. Even if vector control measures are acknowledged and enforced by service members, extreme climatic conditions in the field (temperature > 40 °C) and around-the-clock camp watches may be obstacles to a total adherence to those measures, thus increasing the risk of exposure [27]. Military units are also a mobile population. At the end of their mission, they tend to go on leave abroad, including in countries that are home to competent vectors, leading to a risk of importing arboviruses [4]. In its risk assessment published in October 2016, the European Centre for Disease Prevention and Control (ECDC) concluded that the RVF outbreak in Niger* did not pose a new risk to the European Union [28]. One of their conclusive points was that rural areas should not be considered tourist areas and were unlikely to be visited by ordinary travellers. Considering that more than 10,000 service members from 50 countries, including 19 European countries, are deployed in Mali and neighbouring countries, we recommend that future assessments take account of the specific risk presented by deployed European service members [29].

### Risk of Rift Valley fever virus importation to Europe by travellers

In this study, we illustrate, based on real cases, how RVFV could spread from an endemic area to RVF-free areas. Golnar et al. recently underlined that the highest risk of importing RVFV into the United States was associated with infected humans travelling by plane, a risk that could be similar for Europe [30]. International air travel was also implicated in the spread of CHIKV and ZIKV, leading to emerging global health issues. Two of the RVF cases described here were confirmed after their stay in Mali and had travelled, several days after the febrile stage, throughout areas in Europe (Greece, France, Ireland, Ukraine) where competent vectors are present, especially *Aedes albopictus*, *Ae. vexans* and *Culex pipiens* [4,31]. In the 2006 to 2007outbreak of RVF in Kenya, Njenga et al. estimated RVFV viraemia at 10^1.3^–10^7.8^ TCID_50_ per mL (i.e. 10^1.1^–10^7.6^ plaque-forming units (PFU)/mL) [32,33]. Experimental studies to infect mosquitoes and estimate vector competence exposed mosquitoes to a viraemia threshold > 10^4.7^ PFU/mL [33]. Considering these different points, the risk of RVFV importation to Europe by travellers should be considered as significant.

To our knowledge, this is the first time that the RVFV has been detected in whole blood so long after the onset of symptoms (67 days). In our investigation, RVF RNA was detected in whole blood up to 67 days after the onset of symptoms, but not in serum or plasma. This suggests RVFV RNA compartmentalisation in red blood cells, as already observed for WNV and ZIKV [34-37]. This finding enlarges the window for the use of molecular tools for RVF diagnosis, but it also raises the question of the duration of human viraemia and infectiousness. The maximal extent of human infectiousness for RVFV is unknown and because attempts of viral isolation on cell cultures remained unsuccessful, the infectivity of the virus could not be confirmed in our study. However, hosts with low viraemia could also infect vectors and play a role in the spread of arboviruses [38]. The question remains whether our patients were still infectious when they returned to Europe.

## Conclusion

Our article reports a cluster of human cases of RVF in Mali. Despite an efficient active search that confirmed two of four suspected cases, the alert was issued too late to avoid the risk of RVFV being spread. The confirmed cases had already returned from Mali to Europe. Persistence of detection of RVFV in whole blood until 67 days from symptom onset raises the question of its potential prolonged infectivity. Further studies are needed to explore the duration of human infectiousness and the relevance of PCR on whole blood. As a specific population of travellers, service members should be included in future risk assessments by international and European organisations.

## *Erratum

In the original publication, the sentence referring to the RVF outbreak in Niger erroneously referred to Nigeria. This was corrected on 25 February 2019. We apologise for the mistake.

## Supplementary Data

17000 Supplement

## References

[1] World Organisation for Animal Health (OIE). Rift Valley fever (infection with Rift Valley fever virus). In: OIE Terrestrial manual 2016. Paris: OIE; 2016. Available from: http://www.oie.int/fileadmin/Home/eng/Health_standards/tahm/2.01.18_RVF.pdf

[2] Meegan JM Hoogstraal H Moussa MI An epizootic of Rift Valley fever in Egypt in 1977. Vet Rec. 1979;105(6):124-5. 10.1136/vr.105.6.124 505918

[3] Hassan OA Ahlm C Sang R Evander M The 2007 Rift Valley fever outbreak in Sudan. PLoS Negl Trop Dis. 2011;5(9):e1229. 10.1371/journal.pntd.0001229 21980543 PMC3181235

[4] Chevalier V Pépin M Plée L Lancelot R Rift Valley fever--a threat for Europe? Euro Surveill. 2010;15(10):19506. 20403309

[5] Nicholas DE Jacobsen KH Waters NM Risk factors associated with human Rift Valley fever infection: systematic review and meta-analysis. Trop Med Int Health. 2014;19(12):1420-9. 10.1111/tmi.12385 25252137

[6] Bird BH Ksiazek TG Nichol ST Maclachlan NJ Rift Valley fever virus. J Am Vet Med Assoc. 2009;234(7):883-93. 10.2460/javma.234.7.883 19335238

[7] Mansfield KL Banyard AC McElhinney L Johnson N Horton DL Hernández-Triana LM Rift Valley fever virus: A review of diagnosis and vaccination, and implications for emergence in Europe. Vaccine. 2015;33(42):5520-31. 10.1016/j.vaccine.2015.08.020 26296499

[8] Meegan JM Watten RH Laughlin LW Clinical experience with Rift Valley fever in humans during the 1977 Egyptian epizootic. Contrib Epidemiol Biostat. 1981;3:114-23.

[9] Rudolph KE Lessler J Moloney RM Kmush B Cummings DAT Incubation periods of mosquito-borne viral infections: a systematic review. Am J Trop Med Hyg. 2014;90(5):882-91. 10.4269/ajtmh.13-0403 24639305 PMC4015582

[10] McIntosh BM Russell D dos Santos I Gear JH Rift Valley fever in humans in South Africa. S Afr Med J. 1980;58(20):803-6. 7192434

[11] Al-Hazmi M Ayoola EA Abdurahman M Banzal S Ashraf J El-Bushra A Epidemic Rift Valley fever in Saudi Arabia: a clinical study of severe illness in humans. Clin Infect Dis. 2003;36(3):245-52. 10.1086/345671 12539063

[12] Riou O Philippe B Jouan A Coulibaly I Mondo M Digoutte JP [Neurologic and neurosensory forms of Rift Valley fever in Mauritania] Bull Soc Pathol Exot. 1989;82(5):605-10. French. 2633869

[13] World Health Organization (WHO). Rift Valley fever in Niger. Disease outbreak news. Geneva: WHO; 2016. Available from: http://www.who.int/csr/don/29-september-2016-rift-valley-fever-niger/en/

[14] World Health Organization Regional Office for Africa (WHO/Africa). Niamey: Revue après action contre l’épidémie de la fièvre de la Vallée de Rift au Niger. [Niamey: Review after action against the Rift Valley fever epidemic in Niger]. Brazzaville: WHO/Africa; 2017. French. Available from: http://www.afro.who.int/fr/news/niamey-revue-apres-action-contre-lepidemie-de-la-fievre-de-la-vallee-de-rift-au-niger.French

[15] Bird BH Bawiec DA Ksiazek TG Shoemaker TR Nichol ST Highly sensitive and broadly reactive quantitative reverse transcription-PCR assay for high-throughput detection of Rift Valley fever virus. J Clin Microbiol. 2007;45(11):3506-13. 10.1128/JCM.00936-07 17804663 PMC2168471

[16] Grolla A Mehedi M Lindsay R Bosio C Duse A Feldmann H Enhanced detection of Rift Valley fever virus using molecular assays on whole blood samples. J Clin Virol. 2012;54(4):313-7. 10.1016/j.jcv.2012.04.022 22632901 PMC3398164

[17] Haneche F Leparc-Goffart I Simon F Hentzien M Martinez-Pourcher V Caumes E Rift Valley fever in kidney transplant recipient returning from Mali with viral RNA detected in semen up to four months from symptom onset, France, autumn 2015. Euro Surveill. 2016;21(18):30222. 10.2807/1560-7917.ES.2016.21.18.30222 27172608

[18] Peyrefitte CN Pastorino BAM Bessaud M Gravier P Tock F Couissinier-Paris P Dengue type 3 virus, Saint Martin, 2003-2004. Emerg Infect Dis. 2005;11(5):757-61. 10.3201/eid1105.040959 15890134 PMC3320377

[19] Drosten C Göttig S Schilling S Asper M Panning M Schmitz H Rapid detection and quantification of RNA of Ebola and Marburg viruses, Lassa virus, Crimean-Congo hemorrhagic fever virus, Rift Valley fever virus, dengue virus, and yellow fever virus by real-time reverse transcription-PCR. J Clin Microbiol. 2002;40(7):2323-30. 10.1128/JCM.40.7.2323-2330.2002 12089242 PMC120575

[20] Curasson G [Does Rift Valley fever exist in French Sudan?] Bull Soc Pathol Exot. 1934;27:599-602. French.

[21] World Health Organization (WHO). Weekly bulletin on outbreaks and other emergencies: Week 28: 8-14 July 2017. Geneva: WHO; 2017. Available from: http://www.who.int/iris/handle/10665/255895

[22] Subudhi S Dakouo M Sloan A Stein DR Grolla A Jones S Seroprevalence of Rift Valley fever virus antibodies in cattle in Mali, 2005-2014. Am J Trop Med Hyg. 2018;98(3):872-4. 10.4269/ajtmh.17-0841 29363462 PMC5930922

[23] Food and Agriculture Organization of the United Nations (FAO). Rift Valley fever in Niger. Risk assessment. Rome: FAO; 2017. Available from: www.fao.org/3/a-i7055e.pdf

[24] Mondet B Diaïté A Ndione J-A Fall AG Chevalier V Lancelot R Rainfall patterns and population dynamics of Aedes (Aedimorphus) vexans arabiensis, Patton 1905 (Diptera: Culicidae), a potential vector of Rift Valley Fever virus in Senegal. J Vector Ecol. 2005;30(1):102-6. 16007962

[25] Linthicum KJ Davies FG Kairo A Bailey CL Rift Valley fever virus (family Bunyaviridae, genus Phlebovirus). Isolations from Diptera collected during an inter-epizootic period in Kenya. J Hyg (Lond). 1985;95(1):197-209. 10.1017/S0022172400062434 2862206 PMC2129511

[26] Chevalier V Lancelot R Thiongane Y Sall B Diaité A Mondet B Rift Valley fever in small ruminants, Senegal, 2003. Emerg Infect Dis. 2005;11(11):1693-700. 10.3201/eid1111.050193 16318720 PMC3367374

[27] Michel R Demoncheaux JP Créach MA Rapp C Simon F Haus-Cheymol R Prevention of infectious diseases during military deployments: a review of the French armed forces strategy. Travel Med Infect Dis. 2014;12(4):330-40. 10.1016/j.tmaid.2014.07.001 25052855

[28] European Centre for Disease Prevention and Control (ECDC). Rapid risk assessment: Outbreak of Rift Valley fever in Niger, 10 October 2016. Stockholm: ECDC; 2016. Available from: http://ecdc.europa.eu/en/publications-data/rapid-risk-assessment-outbreak-rift-valley-fever-niger-10-october-2016

[29] United Nations Multidimensional Integrated Stabilization Mission in Mali - MINUSMA. MINUSMA's personnel. New York: United Nations. [Accessed: 21 Feb 2018]. Available from: https://minusma.unmissions.org/en/personnel

[30] Golnar AJ Kading RC Hamer GL Quantifying the potential pathways and locations of Rift Valley fever virus entry into the United States. Transbound Emerg Dis. 2018;65(1):85-95. 10.1111/tbed.12608 28191786

[31] Brustolin M Talavera S Nuñez A Santamaría C Rivas R Pujol N Rift Valley fever virus and European mosquitoes: vector competence of Culex pipiens and Stegomyia albopicta (= Aedes albopictus). Med Vet Entomol. 2017;31(4):365-72. 10.1111/mve.12254 28782121

[32] Njenga MK Paweska J Wanjala R Rao CY Weiner M Omballa V Using a field quantitative real-time PCR test to rapidly identify highly viremic rift valley fever cases. J Clin Microbiol. 2009;47(4):1166-71. 10.1128/JCM.01905-08 19171680 PMC2668310

[33] Golnar AJ Turell MJ LaBeaud AD Kading RC Hamer GL Predicting the mosquito species and vertebrate species involved in the theoretical transmission of Rift Valley fever virus in the United States. PLoS Negl Trop Dis. 2014;8(9):e3163. 10.1371/journal.pntd.0003163 25211133 PMC4161329

[34] Lustig Y Mendelson E Paran N Melamed S Schwartz E Detection of Zika virus RNA in whole blood of imported Zika virus disease cases up to 2 months after symptom onset, Israel, December 2015 to April 2016. Euro Surveill. 2016;21(26):30269. 10.2807/1560-7917.ES.2016.21.26.30269 27386894

[35] Murray KO Gorchakov R Carlson AR Berry R Lai L Natrajan M Prolonged detection of Zika virus in vaginal secretions and whole blood. Emerg Infect Dis. 2017;23(1):99-101. 10.3201/eid2301.161394 27748649 PMC5176245

[36] Rios M Daniel S Chancey C Hewlett IK Stramer SL West Nile virus adheres to human red blood cells in whole blood. Clin Infect Dis. 2007;45(2):181-6. 10.1086/518850 17578776

[37] Lanteri MC Lee T-H Wen L Kaidarova Z Bravo MD Kiely NE West Nile virus nucleic acid persistence in whole blood months after clearance in plasma: implication for transfusion and transplantation safety. Transfusion. 2014;54(12):3232-41. 10.1111/trf.12764 24965017 PMC4268370

[38] Lord CC Rutledge CR Tabachnick WJ Relationships between host viremia and vector susceptibility for arboviruses. J Med Entomol. 2006;43(3):623-30. 10.1093/jmedent/43.3.623 16739425 PMC2814772

