# Erratum for Euro Surveill. 2019;24(8)

**DOI:** 10.2807/1560-7917.ES.2018.24.9.190228e

**Published:** 2019-02-28

**Authors:** Eurosurveillance editorial team

**Affiliations:** 1 European Centre for Disease Prevention and Control (ECDC), Stockholm, Sweden

In the article ‘Tracking Rift Valley fever: From Mali to Europe and other countries, 2016’, published on 21 February 2019, the sentence referring to the RVF outbreak in Niger erroneously referred to Nigeria. This was corrected on 25 February 2019. We apologise for the mistake.

