# Supplementary material for: Tracking Rift Valley fever: From Mali to Europe and other countries, 2016
Source: Euro Surveill. 2019 Feb 21;24(8):1800213. doi: 10.2807/1560-7917.ES.2019.24.8.1800213 (PMC6446957; doi:10.2807/1560-7917.ES.2019.24.8.1800213)
Supplement: Supplement [file 18-00213_POMMIER_Supplement.pdf]

Supplement: Questionnaire administred for Rift Valley fever investigation, Mali-France, 2016

This supplementary material is hosted by Eurosurveillance as supporting information alongside the article [Tracking Rift Valley fever: From Mali to Europe and other countries, 2016] on behalf of the authors who remain responsible for the accuracy and appropriateness of the content. The same standards for ethics, copyright, attributions and permissions as for the article apply.

A) Preceding exposures to Rift Valley Fever Virus

Have you ever been on a trip or on military deployment to Africa or Middle East before?

☐ No ☐ Yes

B) Stay in Mali

Arrival in Mali ☐☐ / ☐☐ / ☐☐☐☐

Departure in Mali ☐☐ / ☐☐ / ☐☐☐☐

From september to your departure, did you stay at Abeibara?

☐ No ☐ Yes

From september to your departure, did you stay in another location in Mali ?

☐ No ☐ Yes

If yes, which ones?

Location 1 ☐☐☐☐☐☐☐☐☐☐☐☐

Location 2 ☐☐☐☐☐☐☐☐☐☐☐☐

Location 3 ☐☐☐☐☐☐☐☐☐☐☐☐

Location 4 ☐☐☐☐☐☐☐☐☐☐☐☐

C) Health status

From september to your departure, did you have any of these following symptoms?

Fever or fever feeling (episode 1) ☐ No ☐ Yes If yes, when? ☐☐ / ☐☐ / ☐☐☐☐

Fever or fever feeling (episode 2) ☐ No ☐ Yes If yes, when? ☐☐ / ☐☐ / ☐☐☐☐

Headache ☐ No ☐ Yes

Retro-orbital pain ☐ No ☐ Yes

Arthralgia ☐ No ☐ Yes

Myalgia ☐ No ☐ Yes

Nose/gums bleeding ☐ No ☐ Yes

Vision trouble ☐ No ☐ Yes

Diarrhea ☐ No ☐ Yes

Vomiting ☐ No ☐ Yes

Colored urine ☐ No ☐ Yes

Other symptoms ☐ No ☐ Yes

If other symptoms, which ones? ☐☐☐☐☐☐☐☐☐☐☐☐ ☐☐☐☐☐☐☐☐☐☐☐☐ ☐☐☐☐☐☐☐☐☐☐☐☐

If you had some symptoms, did you seek for medical care?

☐ No ☐ Yes

Did anyone else in your unit have similar symptoms at the same time?

☐ No ☐ Yes If yes, how many? ☐☐

D) Exposures to Rift Valley Fever Virus

If you had a least one symptom during september to your departure, please answer the following questions:

<u>During the 10 days before your symptom(s)</u>

Did you see any animals (sheeps, goats, zebus, camels,...) ☐ No ☐ Yes

If yes, which animal(s)? Where were they?

☐☐☐☐☐☐☐☐☐☐ ☐ Around the camp ☐ Inside the camp ☐ Other location

☐☐☐☐☐☐☐☐☐☐ ☐ Around the camp ☐ Inside the camp ☐ Other location

☐☐☐☐☐☐☐☐☐☐ ☐ Around the camp ☐ Inside the camp ☐ Other location

Did you pet/touch/handle an animal? ☐ No ☐ Yes

If yes, which animal(s)? Were they dead or alive?

☐☐☐☐☐☐☐☐☐☐☐☐ ☐ dead ☐ alive

☐☐☐☐☐☐☐☐☐☐☐☐ ☐ dead ☐ alive

☐☐☐☐☐☐☐☐☐☐☐☐ ☐ dead ☐ alive

Did you participate in the slaughter of a local animal? ☐ No ☐ Yes

If yes, which animal(s)? ☐☐☐☐☐☐☐☐☐☐☐☐ ☐☐☐☐☐☐☐☐☐☐☐☐ ☐☐☐☐☐☐☐☐☐☐☐☐

Did you drink any local milk? ☐ No ☐ Yes

If yes, from which animal(s)? Did you boil it before drinking?

☐☐☐☐☐☐☐☐☐☐☐☐ ☐ No ☐ Yes

☐☐☐☐☐☐☐☐☐☐☐☐ ☐ No ☐ Yes

☐☐☐☐☐☐☐☐☐☐☐☐ ☐ No ☐ Yes

Did you eat any local meat? ☐ No ☐ Yes

If yes, from which animal(s)? How was the cooking?

☐☐☐☐☐☐☐☐☐☐☐☐ ☐ rare ☐ medium ☐ well-done

☐☐☐☐☐☐☐☐☐☐☐☐ ☐ rare ☐ medium ☐ well-done

☐☐☐☐☐☐☐☐☐☐☐☐ ☐ rare ☐ medium ☐ well-done

Were you bitten by mosquitoes? ☐ No ☐ Yes

If yes, how often? ☐ 1-2 bites/day ☐ 3-5 bites/day ☐ 5-10 bites/day ☐ >10 bites/day

when? ☐ mostly during the day ☐ mostly during the evening ☐ all day long

Were you bitten by other insects? ☐ No ☐ Yes

If yes, which ones? ☐☐☐☐☐☐☐☐☐☐☐☐ ☐☐☐☐☐☐☐☐☐☐☐☐

Did you clean or sleep in a place which hosted some animals? ☐ No ☐ Yes

Did you provide care to a wounded person? ☐ No ☐ Yes

For everybody, during september to your departure:

Did you see any animals (sheeps, goats, zebus, camels,...) ☐ No ☐ Yes

If yes, which animal(s)? Where were they?

☐☐☐☐☐☐☐☐☐☐ ☐ Around the camp ☐ Inside the camp ☐ Other location

☐☐☐☐☐☐☐☐☐☐ ☐ Around the camp ☐ Inside the camp ☐ Other location

☐☐☐☐☐☐☐☐☐☐ ☐ Around the camp ☐ Inside the camp ☐ Other location

Did you pet/touch/handle an animal? ☐ No ☐ Yes

If yes, which animal(s)? Were they dead or alive?

☐☐☐☐☐☐☐☐☐☐☐☐ ☐ dead ☐ alive

☐☐☐☐☐☐☐☐☐☐☐☐ ☐ dead ☐ alive

☐☐☐☐☐☐☐☐☐☐☐☐ ☐ dead ☐ alive

Did you participate in the slaughter of a local animal? ☐ No ☐ Yes

If yes, which animal(s)? ☐☐☐☐☐☐☐☐☐☐☐☐ ☐☐☐☐☐☐☐☐☐☐☐☐ ☐☐☐☐☐☐☐☐☐☐☐☐

Did you drink any local milk? ☐ No ☐ Yes

If yes, from which animal(s)? Did you boil it before drinking?

☐☐☐☐☐☐☐☐☐☐☐☐ ☐ No ☐ Yes

☐☐☐☐☐☐☐☐☐☐☐☐ ☐ No ☐ Yes

☐☐☐☐☐☐☐☐☐☐☐☐ ☐ No ☐ Yes

Did you eat any local meat? ☐ No ☐ Yes

If yes, from which animal(s)? How was the cooking?

☐☐☐☐☐☐☐☐☐☐☐☐ ☐ rare ☐ medium ☐ well-done

☐☐☐☐☐☐☐☐☐☐☐☐ ☐ rare ☐ medium ☐ well-done

☐☐☐☐☐☐☐☐☐☐☐☐ ☐ rare ☐ medium ☐ well-done

Were you bitten by mosquitoes? ☐ No ☐ Yes

If yes, how often? ☐ 1-2 bites/day
☐ 3-5 bites/day
☐ 5-10 bites/day
☐ >10 bites/day

when? ☐ mostly during the day
☐ mostly during the evening
☐ all day long

Were you bitten by other insects? ☐ No ☐ Yes

If yes, which ones? ☐☐☐☐☐☐☐☐☐☐☐☐ ☐☐☐☐☐☐☐☐☐☐☐☐

Did you clean or sleep in a place which hosted some animals? ☐ No ☐ Yes

Did you provide care to a wounded person? ☐ No ☐ Yes
